# The early experiences of Physician Associate students in the UK: A regional cross-sectional study investigating factors associated with engagement

**DOI:** 10.1371/journal.pone.0232515

**Published:** 2020-05-12

**Authors:** Sarah D. Howarth, Judith Johnson, Helen E. Millott, Jane K. O’Hara

**Affiliations:** 1 Leeds Institute of Medical Education, University of Leeds, Leeds, United Kingdom; 2 School of Psychology, University of Leeds, Leeds, United Kingdom; 3 Yorkshire Quality and Safety Research Group, Bradford Institute for Health Research, Bradford Royal Infirmary, Bradford, United Kingdom; 4 School of Healthcare, University of Leeds, Leeds, United Kingdom; Medical University Innsbruck, AUSTRIA

## Abstract

**Background:**

The number of physician associates (PAs) training and working in the UK has increased over the last few years following the proliferation of postgraduate courses. Understanding early experiences and what impacts on engagement is important if we are to appropriately support this relatively new professional group.

**Methods:**

This paper reports on a cross-sectional analysis of the first year of data from a prospective 10-year longitudinal cohort study. First year PA students (n = 89) were enrolled from five universities in one UK region where the training programmes were less than 2 years old. Data collected were: demographic information, wellbeing, burnout and engagement, expectations, placement experience, performance and caring responsibilities. Pearson’s correlations were used to examine relationships between variables and to select variables for a hierarchical regression analysis to understand which factors were associated with engagement. Descriptive statistics were calculated for questions relating to experience.

**Results:**

The experiences of PA students during their first 3–6 months were mixed. For example, 78.7% of students felt that there were staff on placement they could go to for support, however, 44.8% reported that staff did not know about the role and 61.3% reported that staff did not know what clinical work they should undertake. Regression analysis found that their level of engagement was associated with their perceived career satisfaction, overall well-being, and caring responsibilities.

**Conclusions:**

The support systems required for PAs may need to be examined as results showed that the PA student demographic is different to that of medical students and caring responsibilities are highly associated with engagement. A lack of understanding around the PA role in clinical settings may also need to be addressed in order to better support and develop this workforce.

## Introduction

Faced with significant staff shortages, growing demand and rising costs, healthcare systems internationally have increasingly scrutinised their models of care and the configuration of the workforce they employ. In the UK the development of new roles, like that of the Physician Associate (PA), has been declared vital for the future of the NHS [1]. The PA is a mid-level practitioner trained in the medical model to assess, diagnose and commence treatment under the supervision of a physician. Whilst PAs have been integral to the workforce in the United States since the 1960s, only 450 were reportedly practicing in the UK in 2017 and 600 in 2018 [2–3]. This number is expected to rise dramatically over the coming years.

Thus far, research involving this group in the UK has examined patient satisfaction and jurisdictional boundaries in primary care [4–5], their deployment, integration and contribution across hospital settings [6–7] and their impact on postgraduate medical training [8]. One thread of commonality is that the PA role is not well understood by clinical staff [e.g. 9] and that professional boundaries are malleable and subject to negotiation at the point of service delivery [e.g. 4]. This indicates that it may be challenging for PA students to learn their scope of practice in clinical settings, and for PA educators to prepare students for these situations. In the Yorkshire and Humber region in particular, the role is relatively new or unknown in most NHS settings because the first cohort of approximately 25 PA students did not graduate here until 2017. An additional four university programmes have had students graduating since 2018. Consequently, there have been very few qualified PAs employed in this region. Moreover, the majority of new roles in healthcare have been developed from upscaling existing nursing and allied health roles (e.g. Advanced Nurse Practitioners), whereas the training of PAs is arguably unique. They undertake a bespoke 2-year postgraduate educational programme after completing a health-related undergraduate degree, primarily in biomedical science. This is significant as we do not know how this will inform their experience as a healthcare practitioner or as a postgraduate learner.

**Fig 1:**
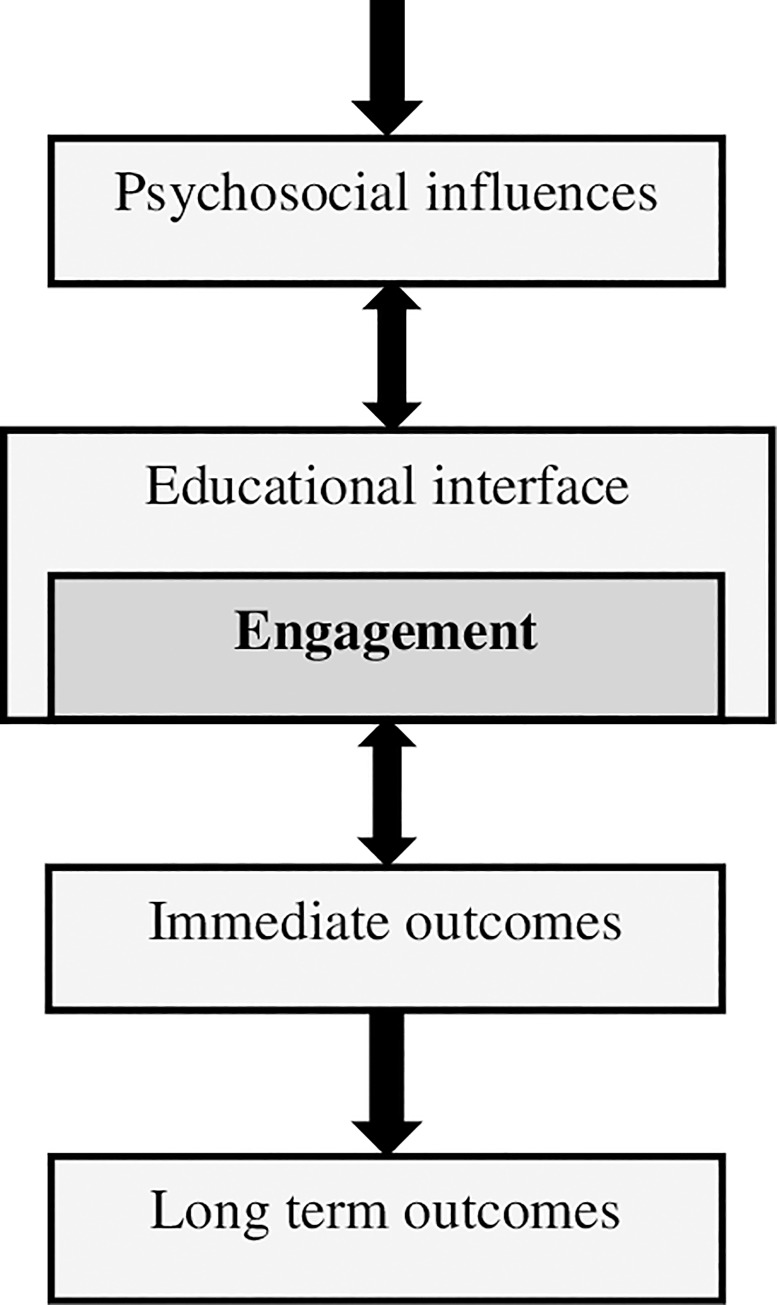
An interpretation of the framework of engagement presented by Kahu and Nelson [16].

Exploring PA student *engagement* is key in this early phase of the development of the discipline. *Engagement* plays a critical role in setting students up for academic success and it is also associated with positive health outcomes [10]. It is particularly important to capture during the early stages of healthcare training, as this is when students are constructing their knowledge of their profession [11] and evidence suggests that it is when they are most at risk of social, health, emotional and financial problems [12–13]. There are many perspectives on how student engagement research should be approached [14]. Kahu’s conceptual framework of engagement [15] and its recent refinement [16] have been widely used [e.g. 17] as a way to develop a more comprehensive understanding of engagement and depict more clearly the mechanisms at work (see Fig 1).

The framework highlights the importance of *structural influences*, such as student background, and *psychosocial influences*, such as motivations and expectations. These are also extensively explored within the wider literature as they define the relationship between each student and the learning environment [18]. Kahu and Nelson [16] suggest that structural and psychosocial influences affect students’ levels of engagement which is conceptualised as a factor comprising emotional, cognitive and behavioural components. Students’ academic self-efficacy, emotions, sense of belonging and wellbeing are also psychosocial constructs but they make up the *educational interface* that engagement operates in. Kahu and Nelson suggest that the *educational interface* contains mechanisms at the intersection of institutional and student factors and that they have the potential to be mediators (i.e. increase or decrease the likelihood of engagement). The level of engagement is then thought to have immediate academic and social outcomes. These operate in a feedback loop with engagement, with more positive immediate outcomes contributing in turn to higher engagement. Finally, the model suggests that immediate outcomes then contribute to long-term outcomes such as retention and personal growth.

Based on this work, we identified two issues that needed addressing presently. First, factors associated with engagement have rarely been assessed collectively in any group of first year healthcare students and so it is unclear which factors may be most important. Second, no engagement research has yet been conducted with PA students. In order to address these issues, the present study investigated factors associated with early engagement in PA students. The study was cross-sectional but occurred within the context of a 10-year longitudinal Physician Associate Cohort Study (PACS), which will comprehensively explore the PA experience from student to practitioner. The study focused on factors which can be viewed as reflecting Kahu and Nelson’s [16]: ‘structural influences’ (caring responsibilities and placement experience), ‘psychosocial influences’ (expectations), ‘educational interface’ (wellbeing and exhaustion) and ‘immediate outcomes’ (performance and career satisfaction).

In summary, the overarching aim of the study was to explore early PA experiences and engagement, in a region where the role is relatively new, in order to be able to better support and train this professional group in the future. More specifically, our two principal research aims were:

- explore the experiences of PA students in their first 3–6 months of training; and,
- identify which factors are associated with greater engagement in this group.

## Methods

### Study design

The present study used a cross-sectional survey design, reporting the first year data from PACS.

**Fig 2:**
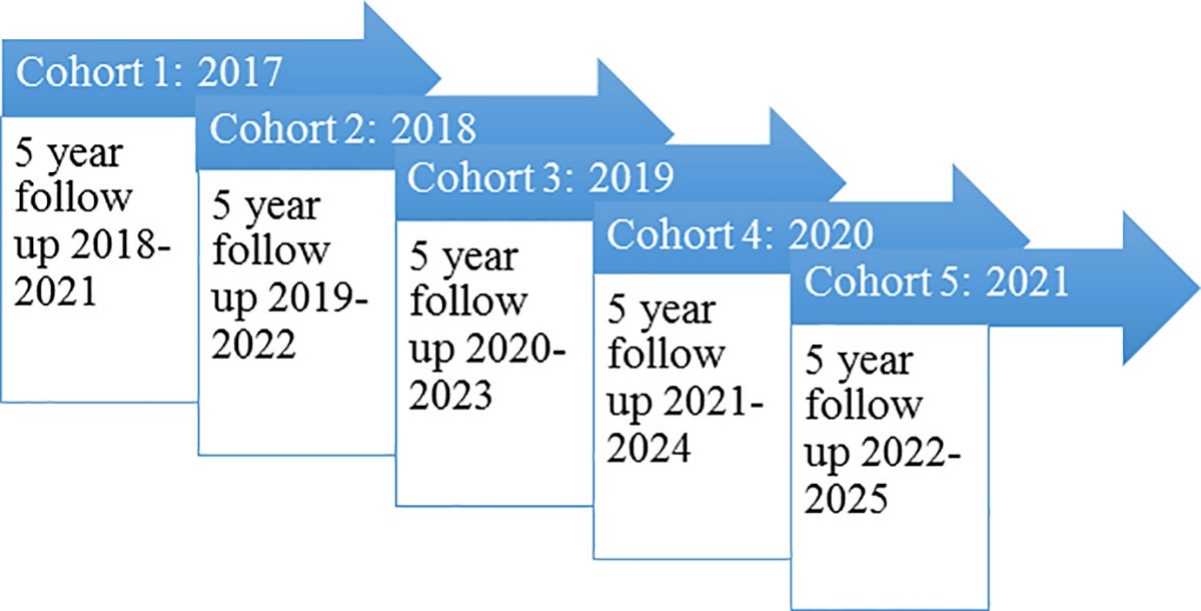
Cohort recruitment and follow-up over 5 years.

PACS is a prospective longitudinal cohort study following five successive years of PA students, beginning at the start of their training, with a follow up of five years (see Fig 2).

### Participants

The present study reported the first year’s data from the first cohort of PACS in order to answer specific research questions. There was a response rate of 74% in year 1 with a total of 89/120 students completing the survey. All students enrolled on PA courses between the academic years 2017 to 2021 (inclusive) at five higher education institutions across the Yorkshire & Humber region will be invited to participate in PACS.

### Measures

The Year 1 PACS questionnaire contains several survey measures. This includes pre-existing measures commonly used in this type of research (e.g. General Health Questionnaire) and measures that have been constructed for this study. These questions were informed by research and reviewed by a Steering Group made up of invited academics and representatives from the institutions involved in PA training. Table 1 presents the measures included in the present study and how they relate to Kahu and Nelson’s Conceptual Framework of Engagement [16].

**Table 1:** Study measures and how they relate to the Conceptual Framework of Engagement.

Measure	Description	Framework of Engagement [16]
**Demographic information**	Age, gender, ethnicity, highest academic attainment and prior healthcare experience.	Structural influences
**General Health Questionnaire (GHQ -12) [19–20]**	This 12-item measure detects minor psychiatric disorders and identifies the severity of any psychological distress experienced over the last few weeks. Each item is scored using a 4-point Likert-type scale from 0 to 3. Sample item: “Have you recently been able to concentrate on what you’re doing?”. This gives a possible total score ranging from 0 to 36, with higher scores indicating more psychological distress (Cronbach’s α = .82). Scores over 12 can be classified as possible cases of psychiatric illness [21–22]	Educational interface/ Immediate outcomes
**Oldenburg Burnout Inventory (OLBI) [23]**	This 16-item questionnaire measures two dimensions: exhaustion and disengagement from work. It was chosen as the scale is both positively and negatively phrased so as to avoid answering bias, which is why it is seen as psychometrically superior to the Maslach Burnout Inventory [24]. Studies have reported its internal consistency is good, the factor structure is robust and test-retest reliability is satisfactory [25]. The items are measured on a 4-point Likert scale with higher scores indicating higher levels of disengagement, or conversely, lower levels of engagement (Cronbach’s α = .70) or exhaustion (α = .80).	**Exhaustion:** Educational interface/ Immediate outcomes
		**Disengagement:** Student engagement
**Expectations and career satisfaction**	Four-items were concerned with expectations around training and employment; one sample item being “so far my expectations for the academic content of the course have been met”. Each item is scored using a 5-point Likert scale. Higher total scores indicate that the student feels positively that expectations are being met (Cronbach’s alpha α = .66). One-item also measured career satisfaction: “I am satisfied with choosing a career as a PA”. This was measured on the same 5-point Likert scale with a higher score indicating more satisfaction.	**Expectations:** Psychosocial influences
		**Career satisfaction:** Immediate outcomes
**Placement experience**	Five-items measured PA student experience whilst on placement using a 5-point Likert scale. Sample item: “other staff knew what the physician associate role is”. Higher total scores indicate a better placement experience (Cronbach’s α = .66).	Structural influences
**Caring responsibilities**	This was measured by 2-items “I have childcare responsibilities for children aged 16 and under” and “I have caring responsibilities for adult relatives/family members” which were scored dichotomously (1-no or 2-yes). A higher total score therefore indicated more caring responsibilities.	Structural influences
**Performance**	Four items measured student perceptions of their academic and clinical performance, such as “I am satisfied with my current performance in clinical work on the physician associate course”. These items were scored using a 5-point Likert scale. Higher scores indicated more satisfaction with their performance (Cronbach’s α = .58). Four items measured performance concerns, such as “I have had concerns raised about my professionalism/fitness to practice” which were scored dichotomously (1-yes or 2-no). Lower total scores indicated more concerns around performance (Cronbach’s α = .89).	**Satisfaction with performance:** Immediate outcomes
		**Performance concerns:** Immediate outcomes

Each follow-up questionnaire in PACS will have some of the same measures (e.g. GHQ-12 and OLBI) and some will be different to reflect the stage of PA training.

### Procedure

Within the first three months of commencing the PA Studies course, students were contacted directly (both face-to-face and via email) with information about PACS. At the end of those 3 months an optional computer session was timetabled at each university and the students in attendance were given the web address and passcode to access the questionnaire on *Online Surveys*. This time period was chosen because students from four of the five universities had some placement experience by then (between 6 and 22 days). The number of placement days varied depending on the setup of the programme (1 primary care day per week or 1 primary care and 1.5 secondary care days per week). For the one university where the students had not been on placement at all, the relevant questions were removed. The questionnaire details were also circulated via email so that they had the option of completing it remotely if desired and a reminder email was sent one month after.

### Data analysis

The number of missing data values was low. There were single missing items on two measures (GHQ-12 0.19%; OLBI 0.002%), which were replaced by the mean of the completed scores for that item across the dataset. As two students did not complete any items on the OLBI measure, these were removed casewise for the purposes of inferential statistics. Six-items measuring placement experience, along with three-items relating to performance and one-item relating to expectations, were not relevant for 16 of the 89 respondents as these students had not yet been on placement at the time of the survey, and so N was adjusted accordingly for both. There was no deviation from normality for all variables.

All data analysis was undertaken using SPSS version 24. In order to address the first aim of the study to investigate participant experiences, descriptive data (means, standard deviations, minimum and maximum values) relating to all variables was calculated and presented. In order to identify associations between the variables, bivariate correlations were are also undertaken using Pearson’s correlation coefficient.

To address the second aim of the study and investigate factors associated with engagement, a hierarchical regression analysis was conducted using the variables which correlated with engagement in the correlation analyses. We entered ‘wellbeing’ into the model first as previous research has suggested it has a strong and consistent association with engagement [26–28] and it sits within the ‘educational interface’ of Kahu and Nelson’s framework [16]. The ‘immediate outcomes’ variables (career satisfaction, satisfaction with performance and performance concerns) were entered next as they are thought to operate in a feedback loop with engagement, and as such could be expected to have a close association. We then added the variable which would be considered a ‘structural influence’ (caring responsibilities), as the model views these as having the most distal contributory relationship to engagement.

### Ethical considerations

The study received ethical approval from the University of Leeds (MREC16-140) and written informed consent was obtained from all participants at the beginning of the survey.

## Results

### Participant descriptive statistics

There was a total of 89 respondents and they ranged in age from 19 to 49 with a mean age of 24.99. The majority were female (71%) with 28% male and 1% other. The majority were also White British (51%) with 22.5% Asian or Asian British–Pakistani, 8% Black or Black British–African and 17 from a range of other ethnicities. To undertake the course, an undergraduate degree in a health-related subject is required, however, 10% also had a postgraduate degree and 1% a doctorate. 58% stated that they had prior experience working in healthcare.

### Research Aim 1. Experiences of first year PA students

Items relating directly to PA student experience were extracted and descriptive statistics conducted (see S1 Table). These suggested that 78.7% and 88.1% of students respectively felt that there were staff on placement that they could go to for support and that they had a good relationship with their supervisor. The majority of students (84.2%) felt that there were not any work-related factors that made their practice unsafe. Yet, 44.8% of students felt that staff did not know about the role and even more staff (61.3%) did not know what clinical work they should undertake. 16.9% said they were finding clinical work difficult and 33.7% said they were finding the academic side of the course difficult.

S2 Table displays the descriptive statistics and the correlations of all variables. These suggested that 44 (49%) participants would be classed as having a possible psychiatric disorder such as minor anxiety or depression. Studies exploring wellbeing in first year medical students found that between 25% and 37% had poor mental health in the first half of the year, which potentially indicates that poor mental health may be more prevalent amongst PA students [29–30]. The means calculated for engagement and exhaustion were 14.78 and 18.96 respectively. This is broadly similar to research undertaken with first year medical students [31].

Wellbeing, career satisfaction, performance satisfaction and caring responsibilities all significantly correlated with engagement (all p < .01), as well as performance concerns (p < .05). These correlations were all in the expected directions and indicated that those who reported more psychological distress and performance concerns; those less satisfied with their career choice and performance; and, those who had more caring responsibilities tended to report being less engaged. Expectations around training and employment and their placement experience were not significantly correlated with engagement.

### Research Aim 2. Factors that predict engagement

To examine which factors were associated with PA student engagement, a hierarchical regression analysis was performed. Variables were entered in 4 steps. Age, gender, ethnicity, highest academic attainment and healthcare experience were input as possible confounding variables. The results can be seen in Table 2.

**Table 2:** Hierarchical regression analysis predicting engagement^a^.

Step	Variable Entered	β	SE β	p-value	Total*R*^2^	Change*R*^2^
1	Age	.047	.062	.452		
	Gender	-.716	.683	.299		
	Ethnicity	.109	.105	.302		
	Healthcare experience	.304	.837	.717		
	Prior Academic achievement	.020	1.021	.984	.043	
2	Age	.032	.055	.560		
	Gender	-.252	.617	.684		
	Ethnicity	.087	.094	.354		
	Healthcare experience	.477	.745	.524		
	Prior Academic achievement	-.255	.911	.780		
	Wellbeing	.279	.065***	.000	.254**	.211
3	Age	.036	.053	.495		
	Gender	-.742	.597	.218		
	Ethnicity	.087	.087	.321		
	Healthcare experience	.255	.704	.718		
	Prior Academic achievement	-.260	.851	.761		
	Wellbeing	.186	.069**	.009		
	Career satisfaction	-1.466	.515*	.006		
	Satisfaction with performance	-.085	.140	.547		
	Performance concerns	-1.178	.687	.091	.386***	.131
4	Age	-.060	.061	.326		
	Gender	-.384	.581	.511		
	Ethnicity	.102	.083	.223		
	Healthcare experience	.263	.668	.696		
	Prior Academic achievement	-.253	.808	.755		
	Wellbeing	.216	.066**	.002		
	Career satisfaction	-1.430	.489**	.005		
	Satisfaction with performance	-.077	.133	.563		
	Performance concerns	-.210	.738	.777		
	Caring responsibilities	2.362	.844**	.007	.455***	.069

*p<0.05

**p<0.01

*** p < .001

^a^Lower scores on this scale indicated higher engagement.

Once the confounding variables were controlled for, wellbeing was significantly associated with engagement F(6,66) = 3.751, p < .01, R^2^ = .254. This model accounted for 21.1% more of the variance than accounted for by the controls. When career satisfaction and satisfaction with performance were added in step 3, the model retained statistical significance F(9,63) = 4.395, p < .001, R^2^ = .386 with an additional 13% of the variance accounted for. Wellbeing and career satisfaction added statistical variance to the model (p < .05), satisfaction with performance (p = .547) and performance concerns (.091) did not. In step 4, caring responsibilities was added and this model was also statistically significant F(10,62) = 5.169, p < .001, R^2^ = .455 with an additional 7% of the variance accounted for. Wellbeing and career satisfaction maintained their significance along with caring responsibilities (p < .01).

## Discussion

Using the first year of data from a 10 year longitudinal cohort study, this paper explored the early experiences of PA students and which factors contributed to their engagement. Higher engagement was associated with greater general wellbeing, higher career satisfaction and not having caring responsibilities. These findings aligned with the conceptual framework of engagement established by Kahu and Nelson [16] in that this study indicated a strong interaction between student engagement and the *educational interface* (wellbeing) and *immediate outcomes* (satisfaction with performance, career satisfaction and performance concerns). The interaction with *structural influences* was mixed with caring responsibilities proving to be significant whilst placement experience was not. In this group, there was no significant interaction found between student engagement and the *psychosocial influences* (expectations around training and employment) which did not align with the conceptual framework [16].

This is the first study to investigate wellbeing and engagement in PAs in the UK. The finding that PA students reported high levels of psychiatric distress and have comparatively poorer mental health than medical students in their first 6 months of training [29–30] is concerning and may indicate that further support is required. There is also reason to expect that these problems may further increase over time, as research has shown that the number of medical students with poor mental health significantly increases over the course of the year [30]. Furthermore, poor mental health has been found to have several ramifications in this group, including impaired academic performance, poorer physical health and reduced self-care practices (e.g. poor diet) [32]. There are, however, significant differences between PA students and medical students, particularly in the design of the programmes. For instance, most PAs are on a clinical placement within their first term (4 out of 5 programmes in the Yorkshire and Humber region) whereas medical students start clinical placements usually from Year 2 or 3. Student wellbeing may then be impacted in very different ways at different stages due to the unique structure of PA Studies programmes.

The level of engagement reported was moderate. When it was high, however, students reported greater general wellbeing, higher career satisfaction and that they did not have caring responsibilities. The finding that wellbeing is linked with engagement is unsurprising and in line with the current literature [33], as is the link between career satisfaction and engagement [34]. The present study extends the existing literature by showing that these factors are also relevant for engagement in PAs. PAs are a new discipline whose remit in the UK is still being defined, with regulation requirements currently under review with the General Medical Council [35]. Ensuring that the job role and career path matches PA student expectations and goals will be important to maintaining the engagement of those in the profession.

One highly significant finding was that PA students with more caring responsibilities were less engaged. Few studies have previously investigated this in other healthcare groups, possibly because these students tend to be undergraduates, and PAs need to be understood as a demographic that is different to that of other healthcare students. PAs are starting the course when they are on average 25 years old, whereas only 7.8% of students are 25 or over when enrolling on undergraduate medical degrees in the UK [36]. The proportion of female PA students is also slightly higher and there is an indication of potentially more ethnic diversity compared with medical students [37]. This suggests that the support systems required may need to be different. Many of the UK-based PA Studies programmes have been set up within medical schools, and there are many advantages to this (e.g. sharing of resources), yet these findings raise important questions around how we can adequately support the individual needs of PA students in ways beyond that provided normally by medical schools. There may also be implications for this in the workplace when supporting PAs once qualified and training in practice too. Lastly, as health services around the world are diversifying their workforce to meet increasing pressures, this work also highlights the importance of recognising the demographic of each professional group as distinct in order to maintain engagement, promote wellbeing and ultimately have a positive impact on patient care.

One interesting finding was that the majority of students found that clinical staff on placement did not know about or fully understand the PA role. Existing research studies have also found this and have argued that it can negatively impact PA integration [9] and restrict PAs in their ability to assume their role [38]. More generally in healthcare this is also not a new phenomenon. Disciplinary tribalism and professional silos have long existed and are a known cause of patient safety incidents [e.g. 39]. However, PA students, certainly those in this region, are in the unusual position of predominantly not being clinically supervised by their own profession. Professional isolation is not currently possible for them in most healthcare settings because very few currently employ qualified PAs. This could be beneficial for breaking down some of the barriers in place in multidisciplinary team working, or it could be an early indication that there will be repercussions if the lack of knowledge around the PA scope of practice is not tackled. More research on this is needed but our findings add to a growing body of evidence suggesting steps should be taken to try and disperse information on the PA role more effectively within clinical settings.

### Limitations

As this paper only reports the first year of data from PACS, it is limited by its cross-sectional design which prevents inferences about the direction of associations. As the PA role is also relatively new and the growth in numbers has been over a short time frame, some of the factors (e.g. average age at enrolment) may change and there will be additional factors impacting PA student experience not captured by this survey. More qualitative research would be useful in unpicking this. Data collection was undertaken within 3–6 months of students starting the course, which meant students had not been on their placements for very long when answering the related questions. Initial conclusions will be strengthened once data has been collected longitudinally. Caring responsibilities proved significant in the analyses but it would have been valuable to stratify between adult and child responsibilities had there been enough power to do this. Whilst pre-existing measures were used (GHQ-12 and OLBI) some of the questions were constructed by the study steering group. It will be important to undertake further validation of these survey measures as we gather data over the cohort period.

## Conclusions

Understanding the early experiences of PA students is important if we are to appropriately support this important new healthcare profession. We found that the experiences of PA students in their first 3–6 months were mixed, with a significant number of healthcare staff perceived to have a lack of understanding about the PA role. This is likely to present a variety of problems for PAs as students and also once they are employed and should be addressed by training programmes and employers. Further, we found that engagement was predicted by career satisfaction, overall well-being, and caring responsibilities. Whilst the cross-sectional design limits inferences in terms of the direction of these associations, it suggests that programmes may benefit from providing additional support to those who have active caring responsibilities, if these students are to continue being engaged members of the PA student community.

## Supporting information

S1 Table Results pertaining to experience-related items. (DOCX)

S2 Table Means, standard deviations and the intercorrelations of all variables. *correlation is significant at the 0.05 level. **correlation is significant at the 0.01 level. ^a^Lower scores on this scale indicated higher engagement.
(DOCX)

S3 Table Data set. (XLSX)

## References

[1] NHS England. NHS Five Year Forward View. 2014 https://www.england.nhs.uk/wp-content/uploads/2014/10/5yfv-web.pdf

[2] Ritsema TS. Faculty of Physician Associates. Faculty of Physician Associates Census results. 2017 https://www.fparcp.co.uk/about-fpa/fpa-census. Accessed 3 Jan 2019.

[3] Ritsema TS. Faculty of Physician Associates. Faculty of Physician Associates Census results. 2018 https://www.fparcp.co.uk/about-fpa/fpa-census. Accessed 3 Jan 2019.

[4] Drennan VM, Gabe J, Halter M, de Lusignan S, Levenson R. Physician associates in primary health care in England: A challenge to professional boundaries? Social Science & Medicine. 2017;181:9–16. 28364578 10.1016/j.socscimed.2017.03.045

[5] Jackson B, Marshall M, Schofield S. Barriers and facilitators to integration of physician associates into the general practice workforce: a grounded theory approach. Br J Gen Pract. 2017;67:e785–91. 10.3399/bjgp17X693113 28993304 PMC5647922

[6] Roberts S, Howarth S, Millott H, Stroud L. ‘What can you do then?’ Integrating new roles into healthcare teams: Regional experience with physician associates. Future Hospital Journal. 2019;6(1):61–6. 10.7861/futurehosp.6-1-61 PMC6520076 31098589

[7] Drennan VM, Halter M, Wheeler C, Nice L, Brearley S, Ennis J, et al What is the contribution of physician associates in hospital care in England? A mixed methods, multiple case study. BMJ open. 2019; 10.1136/bmjopen-2018-027012 30700491 PMC6359738

[8] Roberts S, Howarth S, Millott H, Stroud L. Experience of the impact of physician associates on postgraduate medical training: A mixed methods exploratory study. Clinical Medicine. 2019;19(1):4–10. 10.7861/clinmedicine.19-1-4 30651237 PMC6399627

[9] Halter M, Wheeler C, Drennan VM, de Lusignan S, Grant R, Gabe J, et al Physician associates in England's hospitals: a survey of medical directors exploring current usage and factors affecting recruitment. Clinical Medicine. 2017;17(2):126–131. 10.7861/clinmedicine.17-2-126 28365621 PMC6297628

[10] Christian MS, Slaughter JE. Work engagement: A meta-analytic review and directions for research in an emerging area In: Academy of Management Proceedings 2007, 1 Aug 2007, Briarcliff Manor, NY 10510 Academy of Management 2007:1–6.

[11] Hind M, Norman I, Cooper S, Gill E, Hilton R, Judd P, et al Interprofessional perceptions of health care students. Journal of interprofessional care. 2003;17(1):21–34. 10.1080/1356182021000044120 12772467

[12] McInnis C. Researching the first year experience: where to from here?. Higher Education Research and Development. 2001;20(2):105–114.

[13] Krause KL, Coates H. Students’ engagement in first‐year university. Assessment & Evaluation in Higher Education. 2008;33(5):493–505.

[14] Zepke N. Student engagement research in higher education: questioning an academic orthodoxy. Teaching in Higher Education. 2014;19(6):697–708.

[15] Kahu ER. Framing student engagement in higher education. Studies in higher education. 2013;38(5):758–73.

[16] Kahu ER, Nelson K. Student engagement in the educational interface: understanding the mechanisms of student success. Higher Education Research & Development. 2018;37(1):58–71.

[17] Fisher R, Perényi Á, Birdthistle N. The positive relationship between flipped and blended learning and student engagement, performance and satisfaction. Active Learning in Higher Education. 2018; 10.1177/1469787418801702

[18] Schuetz P. A theory-driven model of community college student engagement. Community College Journal of Research and Practice. 2008;2:305–324.

[19] Goldberg DP. The detection of psychiatric illness by questionnaire. Maudsley monograph. 1972;21.

[20] Goldberg DP. User's guide to the General Health Questionnaire. Windsor. 1988.

[21] Goldberg DP, Gater R, Sartorius N, Ustun TB, Piccinelli M, Gureje O, et al The validity of two versions of the GHQ in the WHO study of mental illness in general health care. Psychological medicine. 1997;27(1):191–7. 10.1017/s0033291796004242 9122299

[22] Liang Y, Wang L, Yin X. The factor structure of the 12-item general health questionnaire (GHQ-12) in young Chinese civil servants. Health and quality of life outcomes. 2016;14(1):136 10.1186/s12955-016-0539-y 27669741 PMC5037881

[23] Demerouti E, Bakker AB, Nachreiner F, Schaufeli WB. The job demands-resources model of burnout. Journal of Applied psychology. 2001;86(3):499 11419809

[24] Maslach C, Jackson SE, Leiter MP, Schaufeli WB. Schwab RL. Maslach burnout inventory (Vol. 21, pp. 3463–3464). Palo Alto, CA: Consulting Psychologists Press 1986.

[25] Halbesleben JR, Demerouti E. The construct validity of an alternative measure of burnout: Investigating the English translation of the Oldenburg Burnout Inventory. Work & Stress. 2005;19(3), 208–220.

[26] Hall L, Johnson J, Watt I, O'Connor D. WellGP: Cross-Sectional Survey of Wellbeing, Burnout, and Patient Safety amongst UK GPs. British Journal of General Practice. 2018 (In press) 10.3399/bjgp19X702713 PMC6592321 31015224

[27] Johnson J, Louch G, Dunning A, Johnson O, Grange A, Reynolds, et al Burnout mediates the association between symptoms of depression and patient safety perceptions: A cross-sectional study in hospital nurses. Journal of Advanced Nursing. 2017;73:1667–1680. 10.1111/jan.13251 28072469

[28] Johnson J, Arezina J, McGuiness A, Culpan A, Hall L. Breaking bad and difficult news in obstetric ultrasound and sonographer burnout: Is training helpful? Ultrasound. 2019;27:55–63. 10.1177/1742271X18816535 30774699 PMC6362539

[29] Guthrie EA, Black D, Shaw CM, Hamilton J, Creed FH, Tomenson B. Embarking upon a medical career: psychological morbidity in first year medical students. Med Educ. 1995;29:337–41. 10.1111/j.1365-2923.1995.tb00022.x 8699970

[30] Moffat KJ, McConnachie A, Ross S, Morrison JM. First-year medical student stress and coping in a problem-based learning medical curriculum. Med Educ. 2004;38:482–91. 10.1046/j.1365-2929.2004.01814.x 15107082

[31] Moffat KJ, McConnachie A, Ross S, Morrison JM. First-year medical student stress and coping in a problem-based learning medical curriculum. Med Educ. 2004;38:482–91. 10.1046/j.1365-2929.2004.01814.x 15107082

[32] Dyrbye LN, Thomas MR, Shanafelt TD. Systematic review of depression, anxiety, and other indicators of psychological distress among US and Canadian medical students. Academic medicine. 2006 4 1;81(4):354–73. 10.1097/00001888-200604000-00009 16565188

[33] Yu L, Shek DT, Zhu X. The influence of personal well-being on learning achievement in university students over time: Mediating or moderating effects of internal and external university engagement. Frontiers in psychology. 2018;8:2287 10.3389/fpsyg.2017.02287 29375421 PMC5767243

[34] Akkermans J, Paradniké K, Van der Heijden BI, De Vos A. The best of both worlds: the role of career adaptability and career competencies in students’ well-being and performance. Frontiers in psychology, 2018; 10.3389/fpsyg.2018.01678 30258381 PMC6143682

[35] House of Lords. The regulation of physician associates (PAs) and anaesthesia associates (AAs). HCWS1741. [Internet. 2019 [cited 2019 July 24]. Available from: https://www.parliament.uk/business/publications/written-questions-answers-statements/written-statement/Commons/2019-07-18/HCWS1741/

[36] Higher Education Statistics Agency (HESA). United Kingdom. 2017. https://www.hesa.ac.uk/data-and-analysis/students/table-6. Accessed 15 Dec 2018.

[37] General Medical Council. 2016. The state of medical education and practice in the UK. https://www.gmc-uk.org/-/media/documents/SOMEP_2016_Full_Report_Lo_Res.pdf_68139324.pdf

[38] Joyce P, Hooker RS, Woodmansee D, Hill AD. Introducing the physician associate role in Ireland: Evaluation of a hospital based pilot project. Journal of Hospital Administration. 2019;8(3):50–60.

[39] Ebert L, Hoffman K, Levett-Jones T, Gilligan C. “They have no idea of what we do or what we know”: Australian graduates' perceptions of working in a health care team. Nurse education in practice. 2014;14(5):544–50. 10.1016/j.nepr.2014.06.005 24999074

