# Supplementary material for: The early experiences of Physician Associate students in the UK: A regional cross-sectional study investigating factors associated with engagement
Source: PLoS One. 2020 May 12;15(5):e0232515. doi: 10.1371/journal.pone.0232515 (PMC7217467; doi:10.1371/journal.pone.0232515)
Supplement: S1 Table — (DOCX) [file pone.0232515.s001.docx]

|  | N | Strongly disagree, n(%) | Disagree, n(%) | Neither agree nor disagree, n(%) | Agree, n(%) | Strongly agree, n(%) | m |
| --- | --- | --- | --- | --- | --- | --- | --- |
| …other staff knew what the PA role is | 73 | 5(6.6) | 29(38.2) | 18(23.7) | 21(27.6) | 3(3.9) | 2.85 |
| …other staff understood what clinical work I should undertake | 72 | 4(5.3) | 42(56) | 15(20) | 12(16) | 2(2.7) | 2.54 |
| …there were other staff I could go to for support | 72 | 1(1.3) | 2(2.7) | 13(17.3) | 44(58.7) | 15(20) | 4.00 |
| …I had a positive relationship with my named supervisor | 73 | 0(0) | 1(1.3) | 8(10.5) | 39(51.3) | 28(36.8) | 4.26 |
| …my practice was not as safe as it could be because of work-related factors or conditions | 73 | 34(44.7) | 30(39.5) | 11(14.5) | 1(1.3) | 0(0) | 1.70 |
| I am finding the clinical work on placement difficult | 73 | 11(13.3) | 32(38.6) | 26(31.3) | 13(15.7) | 1(1.2) | 2.53 |
| I am finding the academic aspect of the course difficult | 89 | 8(9) | 25(28.1) | 26(29.2) | 24(27) | 6(6.7) | 2.94 |
