# Supplementary material for: The early experiences of Physician Associate students in the UK: A regional cross-sectional study investigating factors associated with engagement
Source: PLoS One. 2020 May 12;15(5):e0232515. doi: 10.1371/journal.pone.0232515 (PMC7217467; doi:10.1371/journal.pone.0232515)
Supplement: S2 Table — *correlation is significant at the 0.05 level. **correlation is significant at the 0.01 level. aLower scores on this scale indicated higher engagement. (DOCX) [file pone.0232515.s002.docx]

|  |  | **N** | **Min** | **Max** | **M** | **SD** | **1** | **2** | **3** | **4** | **5** | **6** | **7** | **8** | **9** | **10** | **11** | **12** | **13** | **14** |
| --- | --- | --- | --- | --- | --- | --- | --- | --- | --- | --- | --- | --- | --- | --- | --- | --- | --- | --- | --- | --- |
| **1** | Engagement^a^ | 87 | 8 | 21 | 14.78 | 2.99 | 1 | .099 | -.116 | .125 | .005 | -.001 | **.580**** | **.492**** | -.169 | **-.407**** | -.209 | **-.247*** | **-.398**** | **.309**** |
| **2** | Age | 89 |  |  |  |  |  | 1 | -.016 | -.096 | .276** | -.171 | -.095 | .087 | -.211 | -.141 | .058 | .108 | .112 | .542** |
| **3** | Gender | 89 |  |  |  |  |  |  | 1 | .100 | .031 | -.242* | -.226* | -.132 | -.148 | -.148 | -.016 | -.122 | .098 | -.098 |
| **4** | Ethnicity | 89 |  |  |  |  |  |  |  | 1 | -.007 | -.316** | -.049 | .079 | .015 | .002 | -.063 | .019 | -.060 | -.124 |
| **5** | Highest academic attainment | 89 |  |  |  |  |  |  |  |  | 1 | .027 | -.170 | .072 | -.334** | -.054 | -.039 | -.003 | .065 | .143 |
| **6** | Prior experience in healthcare | 89 |  |  |  |  |  |  |  |  |  | 1 | .052 | -.062 | .042 | -.026 | -.062 | .085 | -.036 | -.086 |
| **7** | Exhaustion | 87 | 8 | 27 | 18.96 | 3.43 |  |  |  |  |  |  | 1 | .568** | .031 | -.143 | -.305** | -.060 | -.404** | .021 |
| **8** | Wellbeing | 89 | 2 | 27 | 11.70 | 4.81 |  |  |  |  |  |  |  | 1 | -.189 | -.242* | -.177 | -.183 | -.456** | .036 |
| **9** | Expectations | 73 | 8 | 20 | 15.15 | 2.65 |  |  |  |  |  |  |  |  | 1 | .578** | .253* | -.078 | .287* | -.103 |
| **10** | Career satisfaction | 89 | 3 | 5 | 4.37 | .63 |  |  |  |  |  |  |  |  |  | 1 | .224 | -.069 | .212 | -.038 |
| **11** | Placement experience | 71 | 13 | 25 | 17.99 | 2.66 |  |  |  |  |  |  |  |  |  |  | 1 | .099 | .319** | -.156 |
| **12** | Performance concerns | 73 | 4 | 8 | 7.90 | .53 |  |  |  |  |  |  |  |  |  |  |  | 1 | .474** | -.365** |
| **13** | Satisfaction with performance | 73 | 6 | 20 | 13.99 | 2.86 |  |  |  |  |  |  |  |  |  |  |  |  | 1 | -.208 |
| **14** | Caring responsibilities | 89 | 2 | 4 | 2.18 | .44 |  |  |  |  |  |  |  |  |  |  |  |  |  | 1 |
